# The Fork sign: a new cortical landmark in the human brain

**DOI:** 10.1093/braincomms/fcae398

**Published:** 2024-11-12

**Authors:** Daniel Kiss-Bodolay, Abdullah Al Awadhi, Karl-Olof Lövblad, Shahan Momjian, Jozsef Zoltan Kiss, Karl Schaller

**Affiliations:** Department of Neurosurgery, Geneva University Hospital, Geneva 1205, Switzerland; Department of Neurosurgery, Geneva University Hospital, Geneva 1205, Switzerland; Department of Radiology, Geneva University Hospital, Geneva 1205, Switzerland; Faculty of Medicine, University of Geneva, Geneva 1211, Switzerland; Department of Neurosurgery, Geneva University Hospital, Geneva 1205, Switzerland; Faculty of Medicine, University of Geneva, Geneva 1211, Switzerland; Faculty of Medicine, University of Geneva, Geneva 1211, Switzerland; Department of Basic Neurosciences, Faculty of Medicine, University of Geneva, Geneva 1211, Switzerland; Department of Neurosurgery, Geneva University Hospital, Geneva 1205, Switzerland; Faculty of Medicine, University of Geneva, Geneva 1211, Switzerland

**Keywords:** human brain, neuroanatomy, cortical landmark, radiological anatomy, anatomical orientation

## Abstract

In the cerebral cortex, establishing the precise relationship between functional areas and the macroscopic anatomy of gyri and sulci has a paramount importance for the field of neuroimaging and neurosurgical interventions. The anatomical orientation should start with the identification of anatomical landmarks to set the anatomo-functional boundaries. The human central sulcus region stands out as a well-defined structural and functional unit housing the primary motor and sensory cortices and is considered as key region to be identified during brain surgery. While useful anatomical landmarks have been discovered, especially in the axial plane, the identification of this region in the sagittal plane remains sometimes difficult. Using cadaveric whole brains and multi-modal analysis of MRI brain scans, we systematically observed a tuning fork–shaped sulco-gyral configuration centred around the gyral continuum bridging the pre-central gyrus with the middle frontal gyrus. We provide evidence that this ‘Fork sign’ is a consistent morphological feature visible on the lateral surface of the brain and a reliable radioanatomical landmark for identifying central sulcus region structures on sagittal MRI images, including the motor hand area.

## Teaser

In this short radioanatomical study, Kiss-Bodolay *et al*. report the identification of the ‘Fork sign’. This new cortical landmark in the human brain is a robust morphological feature visible on the lateral hemispheric surface and a reliable radioanatomical landmark for identifying central sulcus region structures including the motor hand area.

## Graphical Abstract

**Graphical Abstract:**
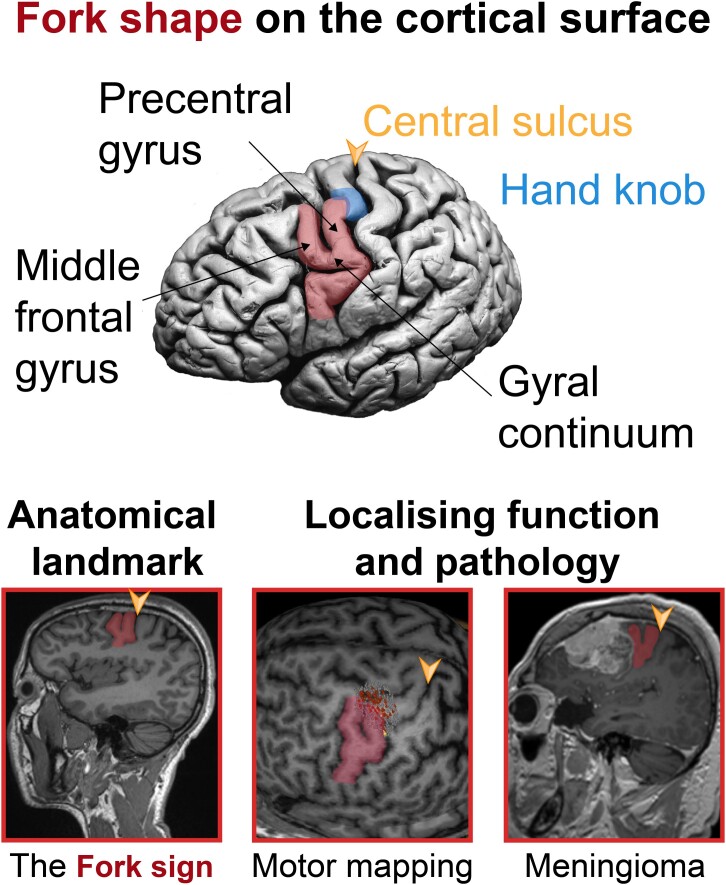

## Introduction

The cerebral cortex consists of functionally specialized subdivisions known as cortical areas.^1^ Defining the relationship between functional characteristics and the macroscopic anatomy of gyri and sulci is of paramount importance for the field of neuroimaging and brain mapping as well as for neurosurgical interventions to predict and prevent postoperative functional deficits.^2-6^ Anatomical orientation in the cortex should start with the identification of known anatomical landmarks to set the anatomo-functional boundaries.^7-11^ The central lobe complex comprising the central sulcus delimited by the pre-central and post-central gyri stands out from the lateral hemispheric surface as an anatomical and functional unit harbouring the primary motor and sensory cortices and is considered as key region to identify during brain surgery.^12,13^ In previous reports, 11 radiological signs based on cortical surface anatomy were described to reliably identify the central lobe.^3,14-21^ While the characteristic anatomy is well described and useful anatomical landmarks have been described, especially in the axial plane, the identification of this region in the sagittal plane remains relatively difficult, and there is clearly a need for more complete knowledge of the sulco-gyral anatomy of this region.^19-21^

We report here a new cortical landmark for reliable identification of central lobe structures in the sagittal plane. We consistently observed a tuning fork–shaped sulco-gyral configuration centred on the gyral continuum running from the pre-central gyrus to the middle frontal gyrus. Based on this morphology, we named it the ‘Fork sign’ that reliably identifies a structural complex composed of the pre-central sulcus and gyrus, the central sulcus, the motor hand area and the intersection between the pre-central and middle frontal gyri.

## Materials and methods

The examination of eight formalin-fixed cadaveric whole brains and eight unpaired hemispheres were carried out by the first author and senior authors in the lab, and metric measurements were done using a flexible graduated rule. After removal of all arachnoid membranes and vessels, both hemispheric surfaces were examined in the region of the central lobe and inspected for the gyral continuum connecting the pre-central gyrus and middle frontal gyrus. To illustrate the anatomo-radiological correlation of the Fork sign, a sagittal cut was made at the level of the insular just lateral to the temporal horn in one hemisphere on which the Fork sign was clear on the convexity surface. Photographs were taken using a standard digital single-lens reflex camera. One hundred hemispheres from 50 MRI brain scans from 50 patients (26 males and 24 females) were analysed retrospectively and included in the data. These patients were admitted for various neurosurgical pathologies including spine and brain lesions and randomly selected from a group of patients with available transcranial magnetic stimulation (TMS) motor mapping. Thirty of them had a pathological hemisphere with a lesion inducing mass effect or oedema reaching the central lobe area. Twenty-three of these patients had bilateral TMS mapping of the motor hand area. Images were analysed using two DICOM viewers (Weasis HUG and BrainLAB AG). Orthogonal planes were analysed using T1 with gadolinium-enhanced images or fluid-attenuated inversion recovery sequences when not available in a minority of cases. For virtual brain dissection, 3D reconstructions of the brain surface could be generated for 42 of the 50 brain scans using an automated segmentation software and inspected for the presence of the gyral continuum between the middle frontal gyrus and pre-central gyrus and the Fork sign on the cortical surface (BrainLAB AG). Three dimensional representation of TMS motor mapping was generated for illustration purposes. Radiological scoring was performed by two observers. After scrolling through the MRI scan, upon agreement, the identification of the radioanatomical sign if present was rated easy, moderate or difficult based on the combination of easiness of agreement and the time necessary to scroll through the image stack. No statistical methods were carried out on the data presented in this work.

### Fork sign

**Figure 1:**
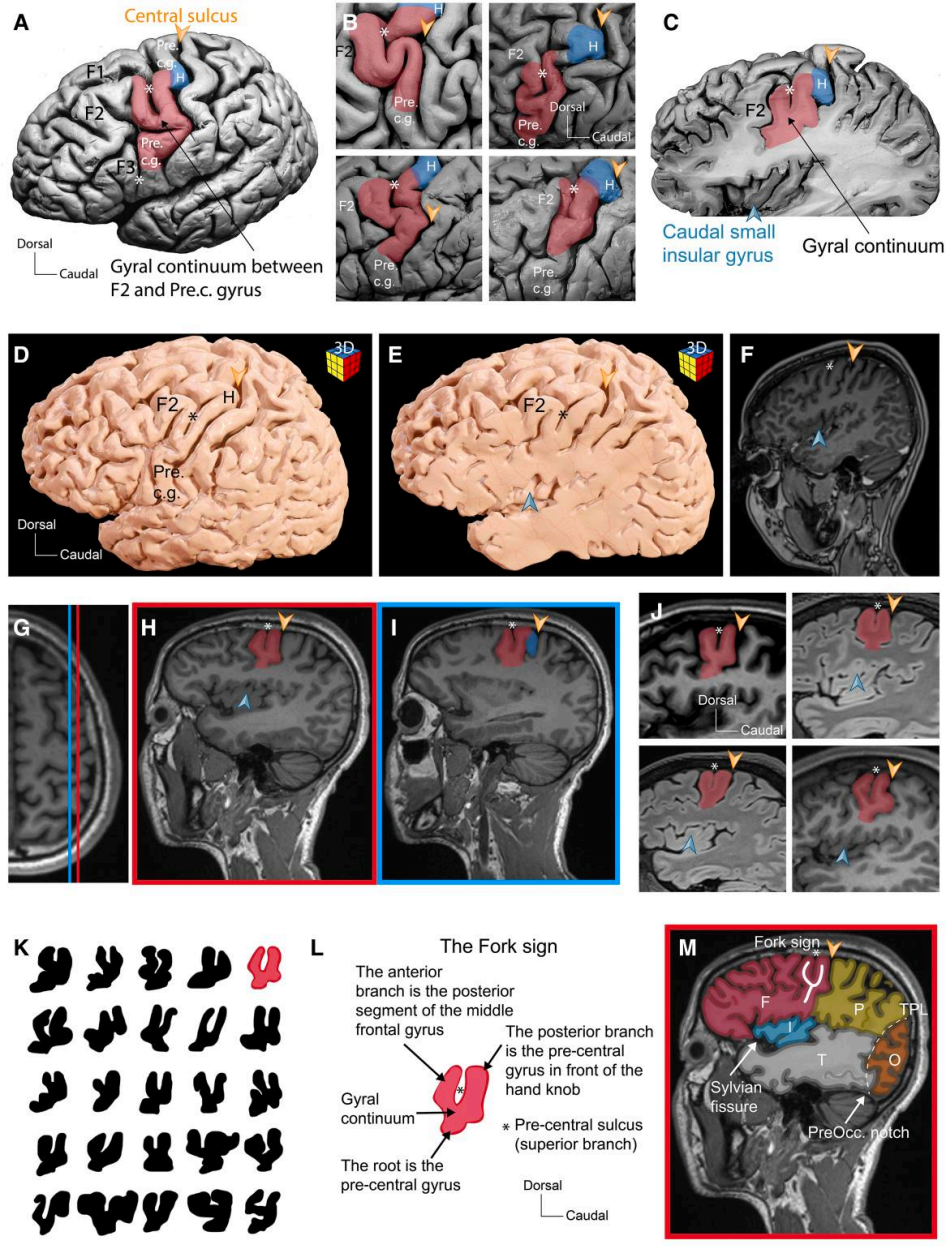
**Anatomy and radiological anatomy of the Fork sign.** (**A**) Lateral view of a cadaveric brain hemisphere illustrating the fork-shaped cortical surface (in red) made up by the middle frontal gyrus (F2), the gyral continuum, and the pre-central gyrus (Pre.c.g.). The hand knob, corresponding to the caudally pointing segment of the upper half of the pre-central gyrus is coloured in blue. (**B**) Representative lateral views illustrating the variability of the fork shape and the hand knob on four cadaveric hemispheres. (**C**) Left hemisphere of a cadaveric brain cut sagittally at the level of the insular cortex cutting through the Fork sign (in red) and hand knob (letter H). The root of the Fork sign points to the caudal small insular gyrus (blue arrowhead). (**D**) Three dimensional reconstruction from a brain MRI scan shows the left hemisphere with the fork-shaped cortical surface. (**E**) Three dimensional reconstruction from the same brain MRI scan as in **D** with a sagittal ‘virtual’ cut at the level of the insula illustrating the presence of the Fork sign. (**F**) Sagittal orthogonal MRI image corresponding to the 3D reconstruction from **E** illustrating the presence of the Fork sign. (**G**) Axial T1-weighted MRI scan indicating the level from which sagittal images in **H** and **I** were taken. (**H**) Sagittal T1-weighted MRI scan at the level of the red line as indicated in **G** illustrating the Fork sign in red. (**I**) Sagittal T1-weighted MRI scan at the level of the blue line as indicated in **G** illustrating the Fork sign in red combined with the Hook sign (letter H). (**J**) Four different sagittal brain MRI scans illustrating the variability of the Fork sign in red. (**K**) Twenty-five different shapes of the Fork sign drawn from 25 different MRI scans (left and right hemispheres). (**L**) Schematic drawing illustrates the typical anatomy of the Fork sign from the example in red from **K**. (**M**) Sagittal brain MRI scan at the level of the Fork sign illustrating the presence of the five lobes in the same plane. The insular lobe (I) is in blue hidden in the depth of the Sylvian fissure. The frontal lobe in red (F) is separated from the parietal lobe (P) by the central sulcus (orange arrowhead) and from the temporal lobe (T) in grey by the Sylvian fissure. The parietal lobe (P) in yellow is separated from the temporal lobe (T) by an imaginary continuity of the Sylvian fissure and from the occipital lobe in orange by the temporo-parietal line (dashed line extending from the projection of the parieto-occipital sulcus to the pre-occipital notch). The occipital lobe (O) in orange is separated by the temporo-occipital line from the temporal (T) and parietal (P) lobes. Asterisk = the pre-central sulcus. Orange arrow head = the central sulcus. The orange arrowhead indicates the central sulcus. The blue arrowhead points to the caudal small insular gyrus. The asterisk indicates the pre-central sulcus. H, hand knob; F1, superior frontal gyrus; F3, inferior frontal gyrus.

**Figure 2:**
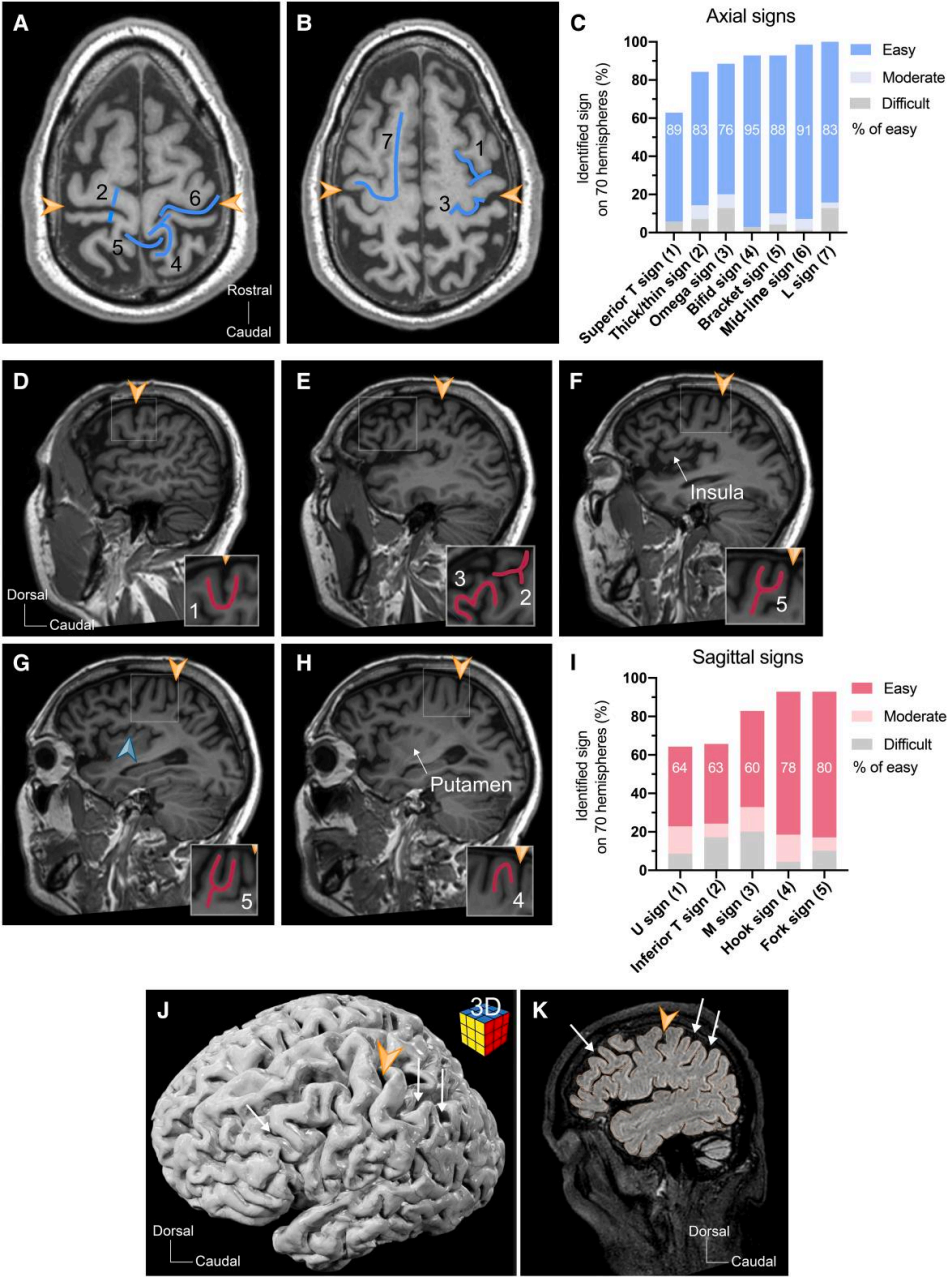
**Robustness of the Fork sign as landmark in healthy hemispheres.** (**A**) Axial MRI plane showing in blue the midline sulcus sign (6), the post-central bifid sign (4), the Bracket sign (5) and the thick/thin sign (2). (**B**) Axial MRI plane showing in blue the L sign (7), the superior T sign (1) and the Omega sign (3). (**C**) Bar plot of frequency (% among 70 hemispheres) and easiness of identification of the axial signs. (**D**) Sagittal MRI plane at the surface of the convexity showing in red the U sign (1). (**E**) Sagittal MRI plane at the level of the temporal lobe’s subcortical white matter showing in red the inferior T sign (2) and the M sign (3). (**F**) Sagittal MRI plane at the level of the insular surface showing in red the Fork sign (5). (**G**) Sagittal MRI plane at the mid-section of the insula showing in red the Fork sign (5). (**H**) Sagittal MRI plane at the level of the medial end of the insula and at the level of the putamen showing the disappearance of the Fork sign and the presence of the Hook sign (4) in red. (**I**) Bar plot of frequency (% among 70 hemispheres) and easiness of identification of the sagittal signs including the Fork sign. (**J**) Three dimensional reconstruction showing a left hemisphere with a corresponding sagittal MRI plane (**K**) illustrating examples of additional U-shaped gyral images surrounding the Fork sign as indicated by the white arrows. The orange arrowhead indicates the central sulcus. The blue arrowhead points to the caudal small insular gyrus.

A Fork sign was scored present if all of the following criteria were met:

- On sagittal planes including the insula, a gyral Y shape pointing upwards was present and faded away at the level of the putamen (Figs 1H, 1I, 1J, 2F and 2G).
- Its caudal branch was in direct continuation with the hand knob when present (Figs 1I, 1J and 2H).
- Its root was directly above or pointed to the most caudal small insular gyrus and was in direct continuity of the peri-insular white matter (Figs 1H, 1I, 1J, 2F and 2G).
- Using multi-planar reconstructions and the rest of the known radiological signs (Fig. 2A, B and D–H), the direct continuity of the rostral branch of the Y with the middle frontal gyrus was established.
- Using multi-planar reconstructions and the rest of the known radiological signs, the direct continuity of the caudal branch of the Y with the pre-central gyrus was confirmed (Fig. 2A, B and D–H).

In addition, seven axial and four sagittal radioanatomical morphology–based cortical landmarks that are daily used in most clinical neuroscience departments were scored on MRI scans using orthogonal sagittal and axial planes in healthy and pathological hemispheres and defined as listed below (Fig. 2A, B and D–H).^2,14-21^

### Sagittal signs

#### U sign

In the sagittal plane, the sub-central gyrus on a far lateral sagittal image takes the form of a U. Often, the pars opercularis can take a U-shaped gyral continuum that can be easily confused with the U sign (Fig. 2D).

#### Inferior T sign

In the sagittal plane, the intersection between the pre-central sulcus and the inferior frontal sulcus takes the shape of a T oriented occipitally. The pre-central gyrus is immediately caudal to the inferior T sign (Fig. 2E).

#### M sign

In the sagittal plane, the gyral root of the orbital and frontal operculi (pars orbitalis, triangularis and opercularis) forms an M-shaped sign in continuity caudally with the pre-central gyrus (Fig. 2E).

#### Hook sign

In the sagittal plane, the hand knob develops into a small downward or ventrally oriented protrusion forming the Hook sign, harbouring the hand knob. This gyral hook is linked to the Fork sign (Fig. 2H).

### Axial signs

#### Superior T sign

In the axial plane, the superior frontal sulcus ends in the pre-central sulcus. When well defined, this intersection forms a caudally oriented T (Fig. 2B).

#### L sign

In the axial plane, the superior frontal gyrus connects to the pre-central gyrus, forming an L-shaped gyral sign oriented outwards (Fig. 2B).

#### Midline sulcus sign

In the axial plane, the central sulcus appears as a long continuous well-defined sulcus perpendicularly oriented to the frontal sulci (Fig. 2A).

#### Omega sign

In the axial plane, the central sulcus takes a U turn around the hand knob towards a caudal direction, forming an Omega sign. Multiple variations of this sign have been described.^22^ We scored the Omega sign as long as a knob was visible (Fig. 2B).

#### Thick/thin sign

In the axial plane, the pre-central gyrus appears thicker than the post-central gyrus especially on its segment medial to the hand knob (Fig. 2A).

#### Bracket sign

In the axial plane, the marginal rami of the cingulate sulci emerge from the inter-hemispheric fissure forming the shape of a bracket concave in a rostral direction (Fig. 2A).

#### Bifid post-central gyrus sign

In the axial plane, the medial terminal segment of the post-central gyrus is often bifid, harbouring the Bracket sign (Fig. 2A).

## Results

### Anatomical and radiological definition of the Fork sign

When examined on the lateral surface of 24 cadaveric hemispheres, the morphology of the central lobe complex, including the dorsoventrally oriented pre-central gyrus, central sulcus and post-central gyrus, can easily be identified (Fig. 1A). The pre-central gyrus harbouring the primary motor cortex is separated anteriorly from the superior (F1), middle (F2) and inferior (F3) frontal gyri by the pre-central sulcus (Fig. 1A). Focusing on the intersection of the pre-central gyrus and the middle frontal gyrus (F2), we consistently identified a gyral continuum bridging the middle frontal gyrus with the pre-central gyrus in all cases (Fig. 1A and B). The position of this gyral passage compared with the hand knob area is shown in Fig. 1A–C. The estimated mean distance between inferior border of this gyral passage and the Sylvian fissure was 3.6 cm (3/6 min/max). We observed that a complex of four structures, the caudal border of F2, the connecting gyral continuum, the pre-central gyrus above this gyral connexion ending at the level of the hand knob and the pre-central gyrus below the gyral connexion takes a tuning fork shape (Fig. 1A). When cut on sagittal planes containing the insular cortex, this gyral configuration is clearly identifiable and still takes a fork shape (Fig. 1C). In addition to cadaveric anatomical observations, we could perform ‘virtual dissection’ of 42 of the 50 brain scans (see the ‘Materials and methods’ section; Fig. 1D and E). We confirmed the presence of a gyral continuum between F2 and the pre-central gyrus in 95% (40/42 brains) of the brains and the presence of a fork shape on the cortical surface in 85% of them (36/42 brains; Fig. 1D and E). When present, this particular cortical arrangement systematically presented the typical fork shape when inspected on an orthogonal sagittal cut (Fig. 1F).

Importantly, the fork-shaped gyral structure and its combination with the hand knob are easily identifiable on sagittal MRI planes cutting through the insula (Fig. 1G–J). Although we observed multiple morphological variations (Fig. 1K), this fork-shaped configuration systematically indicates the position of the middle frontal gyrus and the pre-central gyrus together with the motor hand knob behind it. We named this radioanatomical and cortical surface landmark the ‘Fork sign’ (Fig. 1L). In addition, sagittal planes containing the insula become a powerful anatomical navigation tools after identifying the Fork sign, as the five main lobes can be localized with ease by using the known anatomical boundaries (Fig. 1M). This is not achievable with axial or coronal images.

### Robustness of the Fork sign as radiological landmark

Next, we evaluated the robustness of the Fork sign as landmark and compared it with the already described and used radioanatomical landmarks in MRI images. Two observers scored the 7 known axial signs and 4 sagittal signs in 70 non-pathological and 30 pathological hemispheres (see the ‘Materials and methods’ section and Fig. 2A, B and D–H). The detection rate of the Fork sign was about 93% and easy to identify in 80% of cases, comparable with that of the most used previously identified signs such as the Hook sign on sagittal planes (93% of prevalence and easy in 78%) or the L sign (100% of prevalence and easy in 83%) and the Omega sign (89% of prevalence and easy in 76%) on axial planes (Fig. 2C and I). When present, the Fork sign consistently led to the correct identification of the pre-central gyrus and central sulcus. When the Fork sign was absent, we often observed the presence of an interrupted pre-central gyrus and the presence of multiple gyral connexions between F2 and the pre-central gyrus.

Sagittal MRI images in Fig. 2D–H clearly illustrate that the fork sign is visible and useful landmark only in section planes at the level of the insula. Outside of this level, we observed additional U-shaped gyral variations in more than half of the brain scans that, however, could be easily distinguished from the Fork sign (Fig. 2J and K). Identifying those additional variations helped in correctly scoring the Fork sign. A rostral one constituted by the rostral part of the middle frontal gyrus and a small descending ramus of the superior frontal sulcus, typically pointing 60–90° frontally with no continuity between its root and the peri-insular white matter. A post-central one made up by the post-central sulcus and a small ramus of the post-central sulcus appearing only medially and not overlapping with the sagittal planes of the insula. An angular one made by the angular gyrus and clearly distant from the peri-central area. Very rarely, in cases when the superior branch of the pre-central sulcus descends close to the Sylvian fissure, an ‘early’ Fork sign is visible on sagittal planes laterally to the insula.

**Figure 3:**
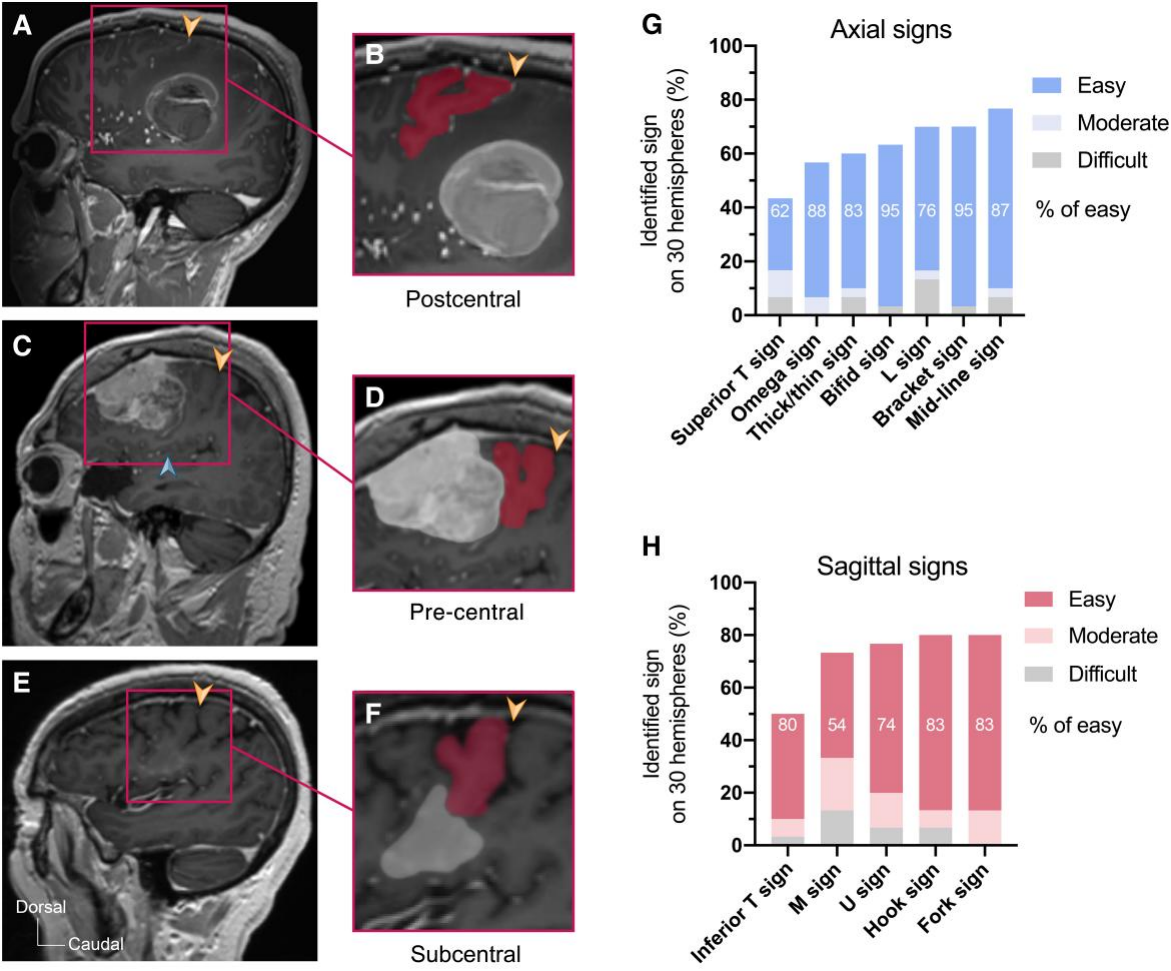
**Robustness of the Fork sign as landmark in pathological hemispheres.** (**A** and **B**) Orthogonal sagittal T1 contrast–weighted MRI scan showing the Fork sign (red area) and an intra-parenchymal parietal haematoma (contrast enhancing and white area). (**C** and **D**) Orthogonal sagittal T1 contrast–weighted MRI scan showing the Fork sign (red area) and a frontal convexity meningioma (contrast enhancing and white area). (**E** and **F**) Orthogonal sagittal T1 contrast–weighted MRI scan showing the Fork sign (red area) and a sub-central glioma (contrast enhancing and white area). (**G**) Bar plot of frequency (% among 30 pathological hemispheres) and easiness of identification of the axial signs. (**H**) Bar plot of frequency (% among 30 pathological hemispheres) and easiness of identification of the sagittal signs including the Fork sign. The orange arrowhead indicates the central sulcus. The blue arrowhead shows the position of the caudal small insular gyrus.

Among the 30 pathological hemispheres, 10 harboured a pre-central lesion (Fig. 3C and D), 2 a post-central lesion (Fig. 3A and B), 4 a sub-central opercular lesion (Fig. 3E and F), 4 a paracentral inter-hemispheric lesion, 5 a parietal lesion, 2 an insular lesion, 2 a parasagittal lesion and 1 harboured a diffuse dural pathology with mass effect. The Fork sign and Hook sign were equally the most frequently preserved and the easiest sagittal landmark to identify in 80% of pathological hemispheres and 83% of cases, respectively (Fig. 3H). In comparison, on axial planes, the Midline sign was the most prevalent (77% of prevalence and easy in 87%), and the Bracket sign was the easiest to identify (70% of prevalence and easy in 95%; Fig. 3G).

### The Fork sign reliably identifies the TMS-mapped motor representation of the hand

**Figure 4:**
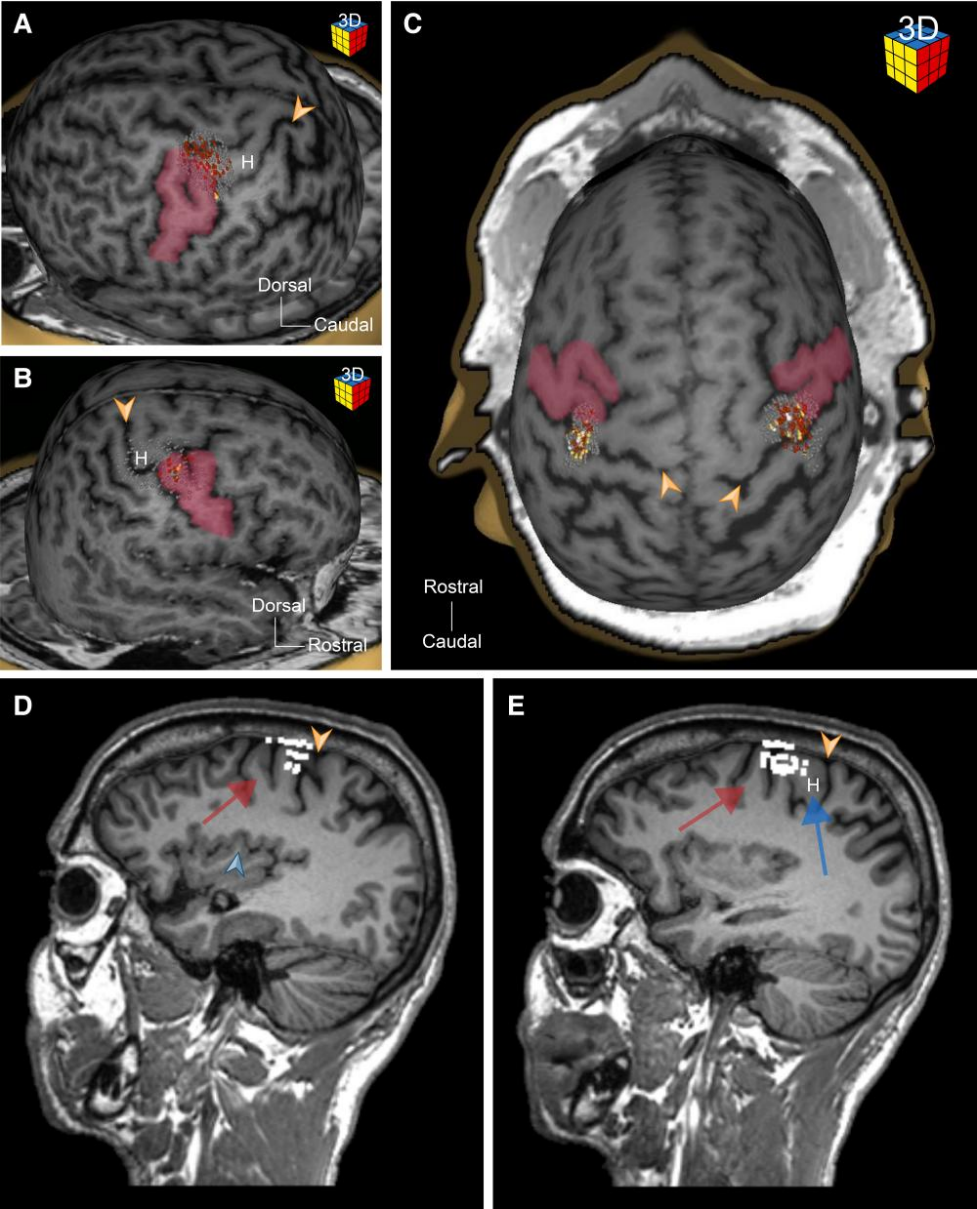
**The Fork sign localizes accurately the TMS-mapped motor hand area.** (**A–C**) Three dimensional object generated based on T1-weighted MRI scan illustrating the TMS-mapped motor hand area (small red and yellow dots) overlapping with the caudal branch of the Fork sign (red area). (**D** and **E**) Sagittal T1-weighted MRI scans illustrating the accuracy at which the Fork sign leads to the localization of the TMS-mapped motor hand area by demonstrating clear overlap between the functional mapping (white dots) and the Fork sign (red arrow) and the Hook sign (blue arrow). The hand knob is indicated by the letter H. The orange arrowhead indicates the central sulcus.

In order to assess the utility of the Fork sign in identifying the motor representation of the hand, we analysed reconstructions and orthogonal MRI images harbouring TMS-based hand motor mapping and applied a systematic approach. Twenty-three patients had TMS hand motor mapping summing up to 24 examined hemispheres. After identifying the Fork sign, its caudal branch was followed to localize the putative motor hand area. In all cases where the Fork sign could be identified, following its caudal branch led to the correct identification of the mapped motor hand area (Fig. 4A–D). Among the 24 mapped hemispheres, 7 showed an overlap of the motor hand map with the caudal branch of the Fork sign rostral to the hand knob (Fig. 4D and E).

## Discussion

Despite the integration of image guidance into clinical neuroscience routines for ∼2 decades, a profound understanding of anatomy and orientation remains pivotal in practice and education.^8,10^ Focusing on the sulco-gyral anatomy of the anterior region of the pre-central gyrus, we systematically observed a gyral continuum linking the pre-central gyrus to the middle frontal gyrus (Fig. 1A, B and D). This finding is consistent with previous descriptions of the pre-central sulcus complex representing the anterior border of the pre-central gyrus.^8,23-25^ The narrow gyral passage between the pre-central and middle frontal gyrus was shown to divide the pre-central sulcus into a superior and an inferior parts.^24^ We could show that this gyral continuum along with neighbouring segments of the pre-central gyrus and the middle frontal gyrus form a tuning fork–shaped complex on the hemispheric surface and in the sagittal plane (Fig. 1A–F). We demonstrate that the Fork sign allows the topographic localization of the pre-central gyrus and the motor hand knob on sagittal brain sections and MRI scans with high accuracy and reliability (Fig. 2). Moreover, this cortical landmark appears easily discernible on the cortical surface, thus potentially allowing the neurosurgeon to locate during operation the corresponding functional areas by exploring the cortical surface (Fig. 1A, B and D).

On MRI scans, the Fork sign appears on sagittal planes containing the insula. If the Fork sign is visible earlier on more lateral planes, usually it is due to an F2-pre-central gyral continuum that is close to the Sylvian fissure (<3 cm from the fissure). We do not consider this image as the true Fork sign. The Fork sign disappears in more medially positioned sagittal planes including central core nuclei such as the putamen. We have demonstrated that the Fork sign is identifiable on sagittal planes with similar frequency to the Hook sign on both healthy and pathological hemispheres (Figs 2 and 3). In comparison with the axial signs, on healthy hemispheres, only the midline sulcus sign and L sign were more prevalent (Fig. 2). This makes the Fork sign, along with the Hook sign, an excellent sagittal radioanatomical landmark on healthy hemispheres and both the most prevalent one among all signs on pathological images even when mass effect or oedema secondary to a lesion affects the central lobe (Fig. 3).

Following a sagittal orthogonal plane at the level of the insular lobe, the posterior ramus of the Fork sign is directly continuous with the Hand knob or so-called Hook sign on MRI scans (Fig. 1A–I).^2^ This observation has been confirmed by the localization of the motor hand region in functional TMS-based motor mapping (Fig. 4). Most of these TMS motor maps were overlapping with the caudal branch of the Fork sign, corresponding to the pre-central gyrus area, outside of the hand knob (Fig. 4D and E). Based on these observations, the Fork sign appears to be an excellent cortical landmark to localize the putative primary motor area even in the absence of functional mapping.

Classically, axial planes are more often used to localize tumours because of the frequency and easiness to reliably identify axial radiological landmarks.^13-21^ Nevertheless, many tumours are localized close to the peri-sylvian area, away from the level of the vertex, and at this level, radioanatomical signs defined on axial planes disappear. In our experience, the use of the sagittal plane to systematically identify the central lobe region appears a better approach, since the Fork sign is easily detectable and the five main lobes (the frontal lobe, parietal lobe, occipital lobe, temporal lobe and insular lobe) are visible together in the same planes that also contain the insula (Fig. 1M).

In addition to the motor representation, the Fork sign, especially its anterior ramus, may also be useful for the localization of other functional areas. Recent studies suggest that cortical areas corresponding to the intersection of the middle frontal gyrus and the pre-central gyrus is critically important for a number of cognitive processes including working memory retrieval,^26^ language processing^27^ and music perception.^28^ Moreover, functional studies^29^ and recent multi-modal parcellation of the cerebral cortex^27^ indicate that this region comprises Area 55B along with the frontal eye field and the pre-motor eye field.^30^ A recent retrospective study of case series^29^ suggests that the posterior middle frontal gyrus, including Area 55b, is an important cortical hub for both dorsal and ventral streams of language. Future studies should clarify the precise relationship between these functional areas and defined segments of the Fork sign complex.

Our study has several limitations. Scoring of the Fork sign and the other known cortical landmarks was based on the evaluation of two raters; thus, we cannot exclude the possibility of subjective bias. The Fork sign may present multiple slight variations, which could demand a certain learning curve before efficient identification. Clearly, the efficient use of the Fork sign requires knowledge of the sulco-gyral anatomy of the peri-sylvian and central lobe regions. We, therefore, advise to use the Fork sign in combination with the other well-described cortical landmarks.

## Data Availability

The MRI data used to investigate our research questions are only available upon special request and in accordance with current legislation since these are patient data.
